# A Mathematical Model of Collective Cell Migration in a Three-Dimensional, Heterogeneous Environment

**DOI:** 10.1371/journal.pone.0122799

**Published:** 2015-04-13

**Authors:** David P. Stonko, Lathiena Manning, Michelle Starz-Gaiano, Bradford E. Peercy

**Affiliations:** 1 Department of Mathematics and Statistics, University of Maryland Baltimore County, MD, USA; 2 Department of Biological Sciences, University of Maryland Baltimore County, MD, USA; Queensland University of Technology, AUSTRALIA

## Abstract

Cell migration is essential in animal development, homeostasis, and disease progression, but many questions remain unanswered about how this process is controlled. While many kinds of individual cell movements have been characterized, less effort has been directed towards understanding how clusters of cells migrate collectively through heterogeneous, cellular environments. To explore this, we have focused on the migration of the border cells during Drosophila egg development. In this case, a cluster of different cell types coalesce and traverse as a group between large cells, called nurse cells, in the center of the egg chamber. We have developed a new model for this collective cell migration based on the forces of adhesion, repulsion, migration and stochastic fluctuation to generate the movement of discrete cells. We implement the model using Identical Math Cells, or IMCs. IMCs can each represent one biological cell of the system, or can be aggregated using increased adhesion forces to model the dynamics of larger biological cells. The domain of interest is filled with IMCs, each assigned specific biophysical properties to mimic a diversity of cell types. Using this system, we have successfully simulated the migration of the border cell cluster through an environment filled with larger cells, which represent nurse cells. Interestingly, our simulations suggest that the forces utilized in this model are sufficient to produce behaviors of the cluster that are observed *in vivo*, such as rotation. Our framework was developed to capture a heterogeneous cell population, and our implementation strategy allows for diverse, but precise, initial position specification over a three- dimensional domain. Therefore, we believe that this model will be useful for not only examining aspects of *Drosophila* oogenesis, but also for modeling other two or three-dimensional systems that have multiple cell types and where investigating the forces between cells is of interest.

## Introduction

Cell migration plays essential roles in multicellular animals [1–3]. Embryonic development provides a clear example of the importance of accuracy in migration, as errors in this process can result in birth defects, such as cleft palate. Proper cell migration is also necessary in adults for a functional immune response and tissue repair. Conversely, improper acquisition of cell motility can promote metastatic cancer progression and inflammatory diseases, such as arthritis [2, 4, 5]. Despite the prevalence of cell motility throughout biology and its contributions to disease pathology, it is not entirely known how underlying mechanisms orchestrate cell movements. While study of individual cell migration *in vitro* has provided a strong basis for understanding this process [2, 6, 7], new questions arise upon consideration of cells moving coordinately, or through varied environments. For example, it is not well-known if collectively moving cells must signal to one another during the migratory process, or if they act autonomously. It is also unclear how the forces generated between the cluster and its surroundings result in coordinated movements.

To address these issues, we have focused on a collective cell migration event within the *Drosophila melanogaster* (fruit fly) ovary, called border cell migration. In collective cell migration, different cell types must synchronize their movements to maintain some cell-cell contacts while disrupting others to change positions in space. Other well-characterized examples of collective cell migration include Zebrafish lateral line [8], streams of neural crest cells [9], and some carcinomas [10]. However, investigating this process in Drosophila is advantageous because of the extensive genetic tools available in this organism and because the tissue can be observed directly *in vivo*[11]. The general process of cell movement as well as the most of the corresponding molecular regulators have been shown to be conserved between Drosophila and other organisms [3, 8, 12].

**Fig 1:**
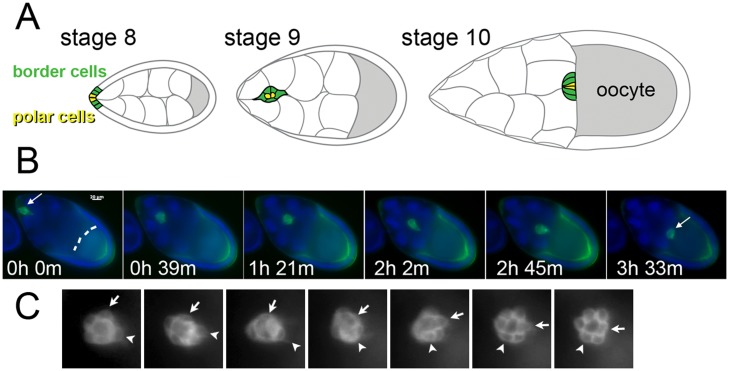
Border cell migration in *Drosophila melanogaster* egg development. (a) At the beginning of stage eight, the polar cells (yellow) and border cells (green) lie in the follicular epithelium of the developing egg chamber. In stage nine, these cells coalesce to form a cluster that detaches from the epithelium. The cluster then translocates between large nurse cells through the egg chamber to reach the developing oocyte (gray) by stage ten. The border cell cluster migrates about 150*μ*m over approximately 4–6 hours. (b) Still images from a time-lapse movie of wild-type border cell migration. The motile cells are marked in green by expression of Slbo-life-Act-GFP. The oocyte, which autofluoresces, is indicated by the dashed line. The nuclei of all cells, including the large, polyploid nurse cells, are seen in blue. In the image at time 0, border cells have already clustered (arrow) and begun moving towards the oocyte. In this example, the border cells reached the oocyte border by 3.5h (arrow on right-most panel). (c) Still images at a higher magnification from a time-lapse movie of a different egg chamber. Images differ by 30 minute intervals. The border cells are marked by a membrane-tethered GFP, and show wild-type behavior. Cells can be observed to change relative positions with respect to the front of the cluster as they move toward the right (arrow and arrowhead indicate the same cell over time). See also Supplemental S1 and S2 Movies.

Genetic and imaging studies have revealed a number of key components in border cell migration. During oogenesis, two polar cells signal to the follicular epithelium at the anterior end of the egg chamber, inducing cell motility. The cells that become motile are the border cells, which coalesce, detach from the epithelium, and move along large, stationary cells called nurse cells. The two polar cells organize into the center of the migratory cluster, where they continuously signal to promote movement, although these cells are not, themselves, motile [13]. The cohort of cells migrate directionally towards the oocyte. This process is depicted in Fig 1. Some growth factors, including certain epidermal growth factors (EGFs) and the platelet-derived/vascular-endothelial growth factor homolog PVF1, are sufficient to attract the border cell cluster, and are produced by the oocyte [3, 8, 14–16]. Interestingly, recent data suggests a mechanical-based tension gradient also contributes to the directed migration of border cells [17]. Border cell migration requires the homophilic adhesion molecule E-cadherin, which provides both the traction needed for movements and strong adhesions to maintain the integrity of the cluster [17, 18]. *In vivo*, the border cell cluster has been shown to rotate or rock while moving, with different cells taking turns as the leading cell [19, 20]. It is not clear if this behavior requires cell-to-cell signaling, or if is an emergent property of the interplay of the physical forces of movement. In addition, while egg chambers with too many or too few border cells do not develop properly, and the number of motile cells is generally consistent [3], it is not know if this number is required due to the physical forces or if it is specified for other reasons. These open questions motivated the construction of a mathematical model to investigate underlying biophysical cell migration mechanisms.

Cell migration modeling has been approached from a number of mathematical perspectives [21–27]. Many investigations have examined how individual cells progress over a flat surface, and are informed by an understanding of molecular dynamics at a sub-cellular level. For example, the general Cytoskeletal Model and Membrane Flow Model arose to capture how actin polymerization leads to extension of the leading edge, and how exocytosis works to progress the cell forward, respectively [28–30]. While these models capture (two-dimensional) changes in a single cell in great detail, they would be computationally expensive to use to model a group of cells moving through a large volume filled with other cells. A classical approach to this type of general modeling problem is a PDE continuum model [31]. However, within the collective cell clusters, neighboring cell types display distinct characteristics. A continuum model does not allow exploration of how varying the cell types or specific number of cells affects the behavior of the system.

Several groups have developed force-based computational models of the movements of cell collectives [24], but none capture all of the features observed for migrating border cells. These frameworks compute the forces of adhesion, repulsion, migration, and stochasticity to determine net cell displacement. Some models nicely recapitulate behaviors of cells in an experimental environment, such as culture media or a matrix [22, 32] but focus on identical types of cells moving over a two-dimensional, uniform surface. A recent agent-based model of Zebrafish lateral line [33] defined multiple cell types, including a non-uniform substrate, but was simulated in two dimensions. Vertex models can reflect morphogenesis well, even in three dimensions, but typically all cells in this type of system have similar properties [34–36]. Three dimensional models to date replicate some observed behaviors of cells moving through an isotropic environment [37, 38], even when paired or clustered [39, 40]. However, the border cell cluster is comprised of cells with different properties, and is known to migrate in a non-uniform environment surrounded by large nurse cells. These differences motivated the construction of a new system to track the positions of cells as they change due to forces from adjacent cells in multiple dimensions, and to capture the heterogeneous cell population that is intrinsic to the developing *Drosophila* egg chamber.

We have developed a new model of the major biophysical interactions between cells in an egg chamber to simulate collective migration through a defined, heterogeneous environment. This force-based mathematical framework captures the collective cell migration process that occurs during stages eight to ten of *Drosophila* oogenesis. We model the cells as spatially discrete using a system of ordinary differential equations, where each cell has a corresponding ODE that tracks the position of that cell in time. We then used this approach to investigate biological hypotheses in the system. In particular, we have investigated whether the defined forces are sufficient to cause cells to switch positions within the cluster as it traverses the egg chamber. We have also examined whether an optimal number of border cells is defined by the biophysical load. Our data suggest that (i) having too few cells in the migratory cluster results in increased migratory time, and that (ii) the four forces in our model (adhesion, repulsion, migration and stochasticity) are sufficient to result in rotation of the migratory cluster, similar to what has been observed. This model may be useful more generally to explore other biological systems where the forces between a diverse cell population are of interest.

## Methods

### Drosophila experiments

Live imaging experiments were performed as described in [41] with the modification of incubating dissected ovarioles in 2*μ*g/mL Hoechst 33342 DNA dye (Invitrogen) for 10 minutes, and then rinsing them gently in dissection media. Images were acquired every 5 minutes for up to 6 hours using a Zeiss AxioImager Z1.1 and AxioVision software. For live imaging, we utilized lines that express membrane-tethered Green Fluorescent Protein specifically in border cells from a transgene. Specifically, we used stocks bearing either the *slbo*-Gal4, UAS-mCD8-GFP [41] or Slbo-lifeAct:GFP [17], which have been shown to have normal cell migration and ovary development. Stocks were maintained at 18 degrees Celsius under standard culture conditions [42]. Flies were fed extra yeast in vials at 29 C overnight, then moved to room temperature for several hours before being dissected. Flies used for imaging egg chambers with differing number of border cells were obtained from Bloomington Stock Center as described in [43].

### Defining Identical Math Cells and minimal forces

To develop a forced-based mathematical model of border cell migration, we chose to model all cells in the tissue as computationally identical cells called Identical Math Cells, or IMCs for short. IMCs have a diameter of 7*μ*m, similar to epithelial cells of the egg chamber, and are discs in two dimensions or spheres in three dimensions. All of the cells experience intrinsic forces from their cytoskeleton and membrane, contact forces from adjacent cells, and small stochastic forces due to Brownian motion. During movement, each cell applies attractive and repulsive forces to adjacent cells. Due to the incompressibility of water/cytosol and the nature of the plasma membrane and cytoskeleton, all of the cells experience repulsive forces in response to contact with neighboring cells. Like a spring being compressed, the further a cell is squeezed, the greater the restoring force is. Outer border cells produce a migratory force, while polar cells do not [3]. Border cells move towards the oocyte due to external cues [3, 8, 14, 15, 17]. The sum of these forces results in a net movement. At the cellular scale, inertia is not relevant.

### Defining a heterogeneous environment using Identical Math Cells

**Fig 2:**
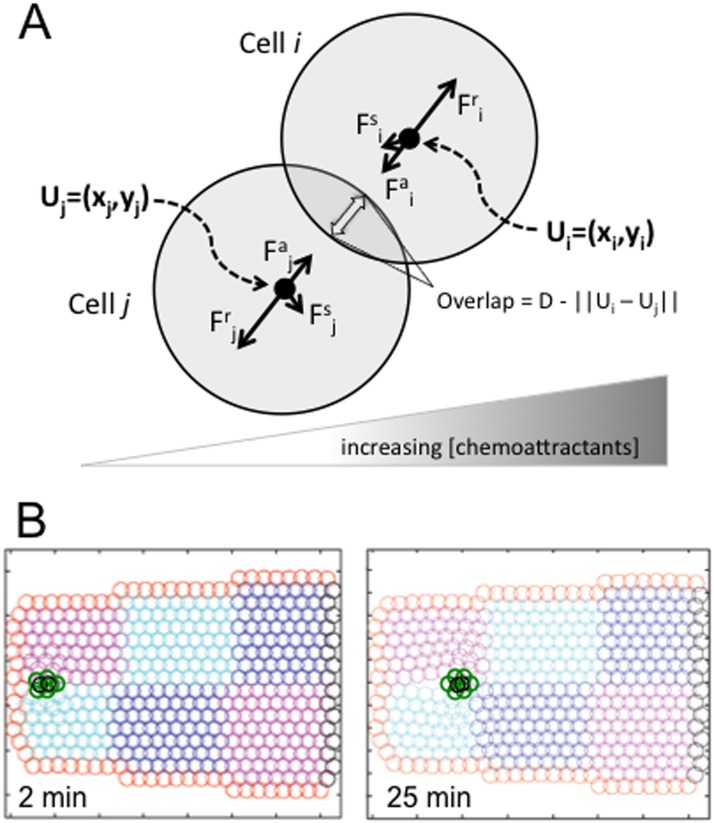
A force-based biophysical model comprised of discrete Individual Math Cells. (a) The forces between two adjacent IMCs, *i* and *j*. The repulsive force acts to separate contacting IMCs, while the adhesive force acts to attract IMC *i* and *j* when they are within an *ε* distance from one another. If one of the IMCs is migratory, it produces a migratory force perpendicular to the axis of interaction due to signal from the gradient of chemoattractants. There is stochastic fluctuation in the position of each IMC. These forces balance and produce overlap between *i* and *j* of *D* − ‖*U*_*i*_ − *U*_*j*_‖_2_, where *D* is the diameter of the IMC. Adhesion force between IMCs creates the integrity of a large single cell or cluster of individual cells with special affinity. The central cluster with a higher adhesion coefficient is closer or more tightly bound to one another than to outside cells, or than outside cells are to one another. (b) The IMC- based domain and simulation in two dimensions. The anterior half of the egg chamber is represented by IMCs with different properties. The epithelial cells are equated to IMCs (red), while the nurse cells are formed from many IMCs (blue, pink, and cyan). The line of black IMCs to the right is the surface of the oocyte at the mid-point of the whole egg chamber. The migratory cluster is formed of tightly bound border cells (green) and polar cells (black). 25 minutes into a 2D simulation, the model shows penetration of the cluster into the egg chamber between malleable nurse cells.

To create a representation of the egg chamber, we constructed a domain filled with IMCs (see Fig 2). We used this construct to model a heterogeneous cell population by assigning distinct properties to the different types of IMCs. Different cell types in the domain have different linear scalars (see below). All of the cells experience repulsive forces due to contact with neighboring cells. Attractive forces are generated between adjacent cells by adhesive forces. We define a basal level of attraction, *β* ^*a*^, which all IMCs feel when they are in close enough proximity to one another. When certain cell types interact, this force is scaled appropriately. The migratory cluster consists of border cells and polar cells, which have very strong adhesive forces between them, representing the high concentration of E-cadherin at these interfaces [3]. We specify the epithelium from the other IMCs in the system by increasing the adhesion between adjacent epithelial cells. This creates a well-defined boundary that acts as the edge of the egg chamber.

Many IMCs aggregated together comprise each large nurse cell. The number of IMCs per nurse cell was chosen to reflect the size difference relative to the epithelial cells. This IMC binning allows interactions between the large nurse cell and smaller border cells to be more localized, and represents intrinsic stiffness of nurse cells created by their complex cytoskeleton and large, polyploid nuclei [11, 44]. Aggregates of IMCs also fill the egg chamber-domain completely, like nurse cells *in vivo*. Within these IMCs, stochastic forces and repulsive forces are maintained to account for the observed integrity and malleability of nurse cells.

### Developing the model in 2D

We utilized a system of ordinary differential equations that track the change in each IMC’s position in time. For example, for the *i* ^th^ IMC, the position of this IMC is *U*_*i*_ = [*x*, *y*]^⊺^. Then, $\frac{dU_{i}}{dt}$ is the change of IMC *i*’s position over time. For each IMC, this change in position is due to the sum of the forces of adhesion from all neighboring IMCs, $F_{i,j}^{a}$, the forces of repulsion from all neighboring IMCs, $F_{i,j}^{r}$, the force of migration, $F_{i,j}^{m}$, and a small stochastic force, $F_{i}^{s}$. Furthermore, the migratory force is generated by IMC *i* if *i* is migratory and is moving past a nonmigratory IMC *j*, but is null if neither *i* nor *j* is migratory, or if they both are migratory because they induce equal and opposite migratory forces. So, for every IMC we have an equation of the form

$$\begin{array}{c} \mu \frac{dU_{i}}{dt}=\sum_{j\in A_{j}}\left(F_{i,j}^{a}+F_{i,j}^{r}\right)+\sum_{j\in M_{j}}F_{i,j}^{m}+F_{i}^{s}, \end{array}$$ (1)

where *A*_*j*_ is the set of all IMCs and *M*_*j*_ is the set of migratory cells. This captures the change of IMC *i*’s position in time, as all IMCs force through adhesion and repulsion with neighboring IMCs, and all migratory IMCs produce a migratory force with *i* by either moving past or pushing into *j*. We define the forces between two IMCs, *i* and *j*, by equations

$$\begin{array}{ccc} F_{i,j}^{a} & = & C_{i,j}^{a}\rho_{i,j}^{\epsilon }H\left(\rho_{i,j}^{\epsilon }\right)\left(d_{i,j}\right), \end{array}$$ (2)

$$\begin{array}{ccc} F_{i,j}^{r} & = & C_{i,j}^{r}{\left(\rho_{i,j}^{0}\right)}^{3}H\left(\rho_{i,j}^{0}\right)(-d_{i,j}), \end{array}$$ (3)

$$\begin{array}{ccc} F_{i,j}^{m} & = & C^{m}\sigma_{i,j}\;{\text{Proj}}_{d_{i,j}^{⊥}}\nabla f, \end{array}$$ (4)

$$\begin{array}{ccc} F_{i}^{s} & = & C^{s}\zeta \left(i\right), \end{array}$$ (5)

where, in two-dimensions, $d_{i,j}^{⊥}$ is the orthogonal subspace to *d*_*i*,*j*_. Here the scalars $C_{i,j}^{a},C_{i,j}^{r},C^{m}$ and *C* ^*s*^ depend on which type cell types are interacting and are detailed later in Methods. *C* ^*m*^ does not have a subscript of *i*, *j* because this interaction is always between a border cell and a non-motile nurse cell. We treat the repulsion coefficient as the same between any two IMCs as well. The adhesion and repulsive forces are governed by the ${\left(\rho_{i,j}^{\varepsilon }\right)}^{k}H(\rho_{i,j}^{\varepsilon })$ term, where *ε* represents the distance beyond the edge of the IMC that the force can act and is 0 for the repulsive force, because cells cannot repel one another if they are not in contact, and *k* = 1 if considering the adhesive force and *k* = 3, inducing nonlinearity, if considering the repulsive force. We assume *ε* > 0 for adherence due to the extension of a protrusion and cell signaling, which we assume to be up to half of the diameter of an IMC (i.e. *ε* < *D*/2). *H*(⋅) is the Heaviside function, which is 1 for a positive argument and 0 for a nonpositive argument. Also, $\rho_{i,j}^{\varepsilon }$ is the overlap of the domains of *i* and *j*, and *d*_*i*,*j*_ is the unit vector from the center of *i* to the center of *j* such that $d_{i,j}=\frac{U_{i}-U_{j}}{\left\|U_{i}-U_{j}\right\|}$ with $d_{i,j}\cdot d_{i,j}^{⊥}=0$. So, the direction of any given adhesive or repulsive force is along ±*d* and the magnitude is given by $\rho_{i,j}^{\varepsilon }H(\rho_{i,j}^{\varepsilon })$ scaled by the appropriate *C* ^*force*^. The migratory force’s sign is determined by *σ*_*i*_ and will be +1 if IMC *i* is migratory and *j* is non-migratory and −1 if IMC *i* is non-migratory and *j* is migratory. Additionally, if IMC *i* and *j* are both non-migratory, or both migratory, then *σ* = 0. This is because non-migratory cells do not produce migratory force, and if two cells are both migratory then the forces are equal and opposite, so they cancel. More specifically, the IMC will produce a migratory force in the direction perpendicular to the axis of interaction. This is described in more detail in the S1 Appendix in the Supplemental Material. Thus, the cells move in response to the chemical gradient in the direction perpendicular to their interaction as they push off one another. Lastly, the stochastic force perturbation on each cell is created by *ζ*(*i*), which is a stochastic force generator that has *x*- and *y*-components taken from a Gaussian distribution with mean zero and unit standard deviation. This force is generated biologically when a cell extends a protrusion and generates a small amount of force in a random direction. Taken together, these four forces characterize the migration of each of the IMCs in time through the set of equations provided in (Eq 1). These interactions are summarized in Fig 2.

### Simulation in 2D

Neither the damping viscosity coefficient nor the IMC interaction forces have been quantified in this fully heterogeneous environment. However, much work has gone into cell-substrate measurements in some similar two-dimensional environments (for examples, see [27, 45–47]), which provides some basis for estimation. Thus, we consider the non-dimensionalized form of the parameters and capture relative strengths of these forces that lead to physiological behavior. (See Table 1). In our early experiments, we non-dimensionalized this system and ran simulations using a forward Euler method due to the stochastic force. The simulation was implemented in Matlab. Our implementation captured many of the dynamics of the system (Fig 2B). We observed maintenance of the overall architecture of the IMC-based egg chamber, translocation of the border cell cluster towards the oocyte, and localization of the two polar cells to the center of the motile cell cluster. The time scale of the process was also similar to that *in vivo*. These results allowed us to fine tune parameters to obtain the most accurate simulation (see below and [48]).

**Table 1:** Composite non-dimensional parameter values used for simulation. The parameter *α* is the time scale (an hour) and *D* is the diameter of an IMC (7*μ*m). We can consider the *α* and *D* scaling the damping viscosity coefficient, *μ*, then the parameters in the table represent a ratio of force exerted on the IMC by movement through the heterogeneous medium and the force exerted by the adhesion, repulsion, migration and stochastic forces.

Parameter	Value
$\frac{\alpha }{D\mu }\beta^{a}M_{B}^{a}$	1.872
$\frac{\alpha }{D\mu }\beta^{a}M_{P}^{a}$	1.872
$\frac{\alpha }{D\mu }\beta^{a}M_{P,B}^{a}$	7.02
$\frac{\alpha }{D\mu }\beta^{a}M_{E}^{a}$	7.02e-4
$\frac{\alpha }{D\mu }\beta^{a}M_{N}^{a}$	7.02e-4
$\frac{\alpha }{D\mu }\beta^{a}M_{B}^{a}$	1.872
$\frac{\alpha }{D\mu }C^{m}$	1.56
$\frac{\alpha }{D\mu }C^{r}$	15.6
$\frac{\alpha }{D\mu }C^{s}$	5

### Implementing the model in 3D

The three-dimensional mathematical model of this system is similar to the two-dimensional model, but the IMC position vector takes the form *U*_*i*_ = [*x*, *y*, *z*]^⊺^. With this slight change, the adhesive, repulsive and stochastic forces are calculated as before (as defined in Eqs (2), (3), and (5), respectively). Epithelial cells are defined at the edges of the domain, other than the posterior side representing the oocyte surface, and aggregated IMCs establish 15 nurse cells, as in *in vivo*. Since it is unnecessary to calculate interactions for IMCs beyond one cell away, we collected set of IMCs with a two-cell-diameter neighborhood for each IMC when calculating the forces and updated this set every 0.2 time units. We implemented this model in Matlab and on UMBC’s HPCF (www.umbc.edu/hpcf/) with a forward Euler step of 0.005 corresponding to approximately 18 seconds. Numerical studies were run to show convergence on the order of the time step without stochastic forces. We also developed a graphical user interface by which initial IMC positions and properties may be specified in planes of IMCs from anterior to posterior [49]. Through this implementation we can simulate the cluster migrating through the egg chamber, which mirrored what has been observed by live imaging (see Results).

### Specification of scalars

The linear scalar $C_{i,j}^{a}$ is a parameter that depends on which two biological cell types are interacting. Due to differences in the biology, these parameters differ from cell type to cell type and must be defined. The corresponding linear scalars for the repulsive and migratory forces are equivalent regardless of cell type. Depending upon cell type, we specify

$$\begin{array}{ccc} C_{i,j}^{a} & = & \left\{ \begin{array}{cc} \beta^{a}M_{B}^{a} & \;\;i\;\text{and}\;j\;\text{are}\;\text{border}\;\text{cells},\\ \beta^{a}M_{P}^{a} & \;\;i\;\text{and}\;j\;\text{are}\;\text{both}\;\text{polar}\;\text{cells},\\ \beta^{a}M_{P,B}^{a} & \;\;i\;\text{or}\;j\;\text{is}\;\text{a}\;\text{polar}\;\text{cell}\;\text{and}\;i\;\text{or}\;j\;\text{is}\;\text{a}\;\text{border}\;\text{cell},\\ \beta^{a}M_{E}^{a} & \;\;i\;\text{and}\;j\;\text{are}\;\text{both}\;\text{epithelial}\;\text{cells},\\ \beta^{a}M_{N}^{a} & \;\;i\;\text{and}\;j\;\text{are}\;\text{part}\;\text{of}\;\text{the}\;\text{same}\;\text{nurse}\;\text{cell},\\ \beta^{a} & \;\;\text{otherwise}. \end{array}\right. \end{array}$$ (6)

Here we scale the adhesive forces based on the types of cells that are interacting. We define a basal level of attraction, *β* ^*a*^, which all IMCs feel when they are in close enough proximity to one another. When certain cell types interact, this force is scaled appropriately, as shown above. In the case of our model, we place strong adhesive forces between interacting epithelial cells. We also maintain very strong adhesive forces between interacting border cells and interacting polar cells [3]. After a brief initial time, we fix the position of the outer epithelial cells to make visualization of the migrating cluster more clear. We can think of this as including a strong extracellular matrix and sheath surrounding the egg chamber, but it is not necessary to maintain the integrity of the chamber in the simulation. We non-dimensionalize the variables and parameters of the system for simulations. The parameter values for the force constants are in Table 1.

## Results

### Simulation from a 2D model reflects the *in vivo* dynamics of a motile cell cluster

We first developed a forced-based mathematical model of border cell migration in two dimensions. Each cell in the system, modeled as an identical math cell (IMC), is subject to to the forces of adhesion, repulsion, migration and stochasticity; the sum of these forces results in a net movement (Fig 2A, and see Methods). All of the cells experience intrinsic forces from their cytoskeleton and membrane, contact forces from adjacent cells, and small stochastic forces due to Brownian motion. However, different cell types can be modeled by designating different linear scalars for different IMCs. Interestingly, only the border cells produce a migratory force, while polar cells are non-motile [3]. Border cells create a migratory force by polymerizing actin in the direction that they are migrating, which pushes the cell forward towards the oocyte in response to directional cues. To do this, motile cells also must push back against the neighboring cells. The border cells thus exert forces on each other and non-moving nurse cells (which are bounded by a follicle cell layer and extracellular matrix that limit their movement). Throughout the migratory process, each cell applies attractive and repulsive forces to adjacent cells. Specifically, cell adhesion molecules, like cadherins, mediate connections between cells, pulling them into closer contact [17, 18, 50], while repulsive forces arise between adjacent cells when the cytosol is compressed.

*In vivo* imaging suggested that migrating border cells must force the large nurse cells out of the way as they move. This deformation is difficult to detect when only border cells are labeled, so we adapted our time-lapse imaging method to label nurse cell nuclei with Hoechst DNA dye (see Methods) and included the membrane label FM4-64 as described in [41]. These movies more clearly reveal the nurse cell dynamics as the border cells squeeze between them (Fig 1B and Supplemental S1 Movie). To represent the large nurse cells in the model, we aggregated enough IMCs to approximate the size of a nurse cell, and used 6 aggregates to represent a cross-section of the environment of the egg chamber (Fig 2B, and see Methods). This IMC binning allows interactions between the large nurse cell and smaller border cells to be more localized, reflecting the character observed for these cells and their structured cytoskeleton [11, 44]. Tightly adhering IMCs bounded the domain to represent the follicular epithelium.

We utilized a system of ordinary differential equations to track the changing positions of each IMC during egg development (see Methods). Simulation displayed maintenance of the overall architecture of the IMC-based egg chamber over time and translocation of the border cell cluster towards the oocyte, which accurately reflected what is observed in live imaging (Fig 2B). Interestingly, the two polar cells localized to the center of the motile cell cluster as seen *in vivo* (Figs 1C and 2B). The time scale of the process was also similar to that *in vivo*. However, the two-dimensional model did not capture all of the processes of interest, such as the rotation of the border cell cluster as it moves (Fig 1C and Supplemental S2 Movie). This property is difficult to ascertain in two dimensions, since, *in vivo*, individual cells move into different planes of the egg chamber. Interest to investigate this phenomena motivated our construction of a three-dimensional model.

### Simulations from 3D model capture cell position-switching within the cluster

We aimed to use the model to test whether the basic forces we defined were sufficient to govern the migration behaviors observed *in vivo*. One interesting phenomenon that occurs during *Drosophila* oogenesis is the tumbling of the border cell cluster as it progresses through the egg chamber, where different cells take turns in leading and lagging positions (see Fig 1C, Supplemental S2 Movie, and [19, 20]). A favored hypothesis to explain this is that chemoattractant receptors saturate in the cells at the front, causing lateral cells to increase activation levels and switch positions. In our model, we only have four forces: adhesion, repulsion, migration and a small stochastic fluctuation. This provided a novel way to assess if the physical forces at play around the cluster are sufficient to recapitulate the dynamics in the absence of a more complex biological mechanism. However, results from the 2D simulation suggested this would be difficult to capture without tracking the third dimension (in and out of the plane). Thus, we implemented our basic model in three dimensions.

**Fig 3:**
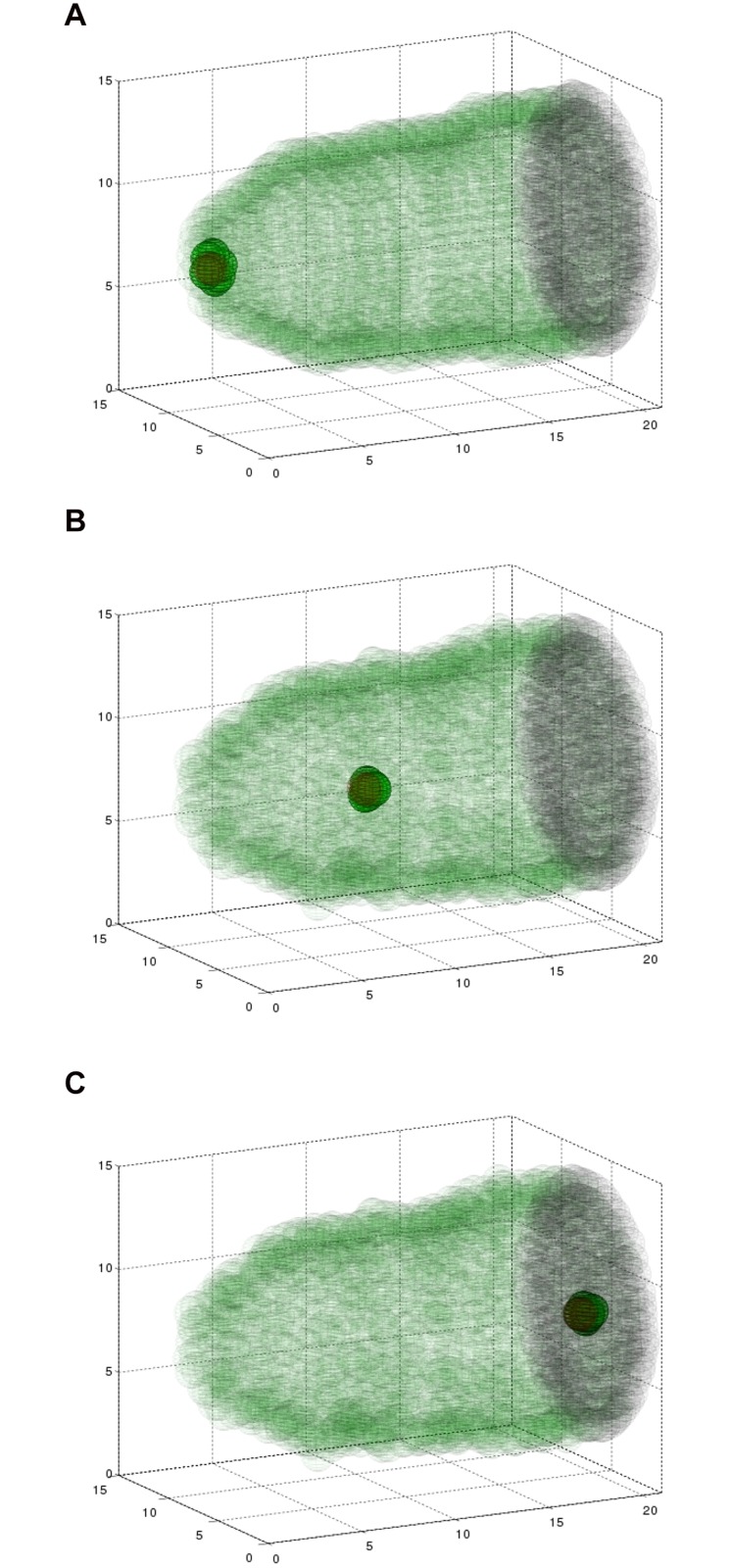
Simulating the three dimensional model results in collective migration. A simulation showing six border cells (green), two polar cells (red), the epithelium (transparent green), and the surface of the oocyte (black, right) at three time points during the migration. Fifteen nurse cells are situated inside the egg chamber, but are not plotted so as to maintain clarity of this three dimensional structure. Polar cells are surrounded by border cells, making them hard to distinguish. (A) At 2 minutes, cells are beginning to invade between nurse cells. (B) At 2.4 hours, the cluster is about halfway to its destination. (C) At 5.6 hours, the border cell cluster has reached the edge of the oocyte. See also Supplemental S3 Movie.

**Fig 4:**
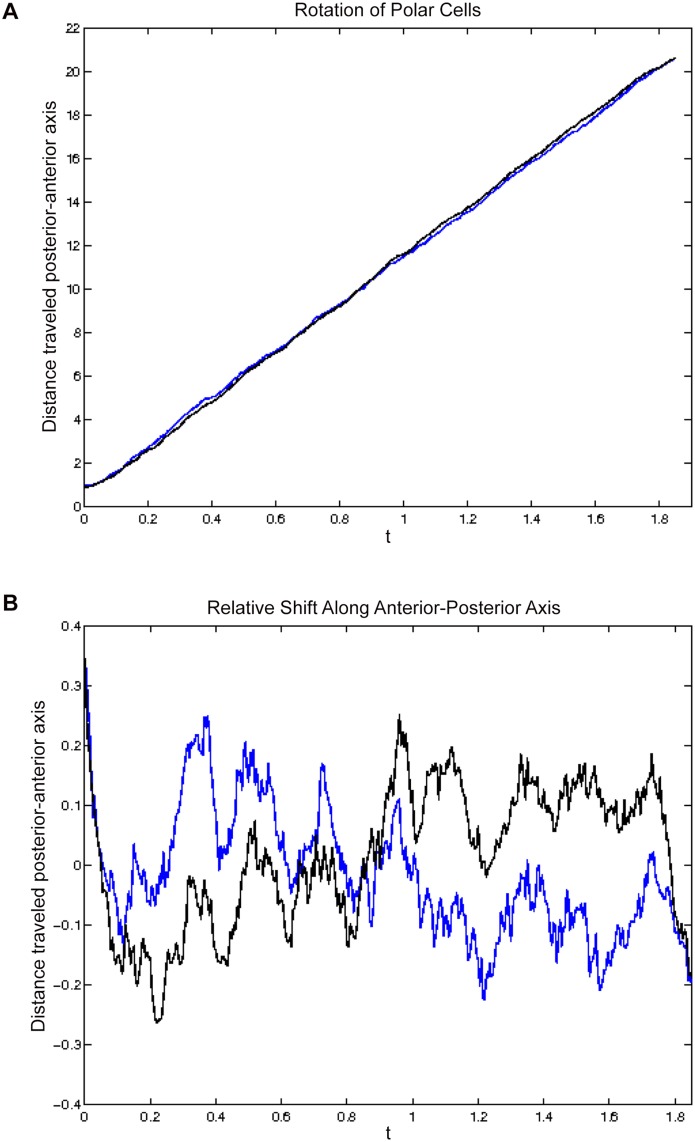
Polar cell positions along main axis of migration. (A) The distance of the polar cells from the anterior of the egg chamber versus time. (B) The relative positions of the two polar cells to one another, along the axis that runs from anterior to posterior through the egg chamber. Each line corresponds to one of the polar cells. As the cluster moves forward, we observe that the polar cells are changing position with respect to one another along this axis, including a complete switch at 0.8 hours. This simulation modeled six border cells.

Through this implementation, we can simulate the cluster migrating through the egg chamber, shown as a time course in Fig 3 (see also, Supplemental S3 Movie). Here, six border cells (green) begin in the epithelium (transparent green) and migrate in the direction of the oocyte (black) carrying the non-motile polar cells (red). Qualitatively, this simulation mirrored what has been observed by live imaging. We observed and measured the position of the migratory IMCs in the 3D simulations (see Methods). First, we observed that by tracking an individual border cell through a complete migration, that in some cases, it did move from the posterior side of the cluster to the anterior side, and vice versa. To quantify the rotation of the interior of the cluster, we tracked the position of each of the polar cells as they move along the direction of the gradient. Fig 4A shows this position versus time for each of the polar cells, with one shown in black and one shown in blue. Fig 4B shows the same data, but the vertical axis represents the relative position of the polar cells to one another. These show that the polar cells switch positions in the cluster with respect to one another several times during this migration. There is one clear switch around *t* = 0.8, as well as a change in position at the beginning. The relative rotation captured in Fig 4B shows a shift of one polar cell leading by a maximum 1.4 *μ*m to the other leading by a maximum of 1.4 *μ*m. This suggests a rotation of about 45° with polar cell diameters of 7*μ*m in about two hours. Fig 1C shows about a 90° rotation over the 3 hour time period. While rotation can certainly be occurring in the full three-dimensions, this tracking along the anterior-posterior axis captures significant generic rotation.

To assess further the impact of the randomness on the process we also ran multiple simulations (N = 10) and calculated statistics on the number of polar cell lead changes and the maximal rotation. We find that the average number of clear transitions where the lead cell switches (movement greater than 0.1 times the cell diameter) is 3.4 times with a range of (1,7) in a two hour period. For each two hour run, we calculate a maximal lead for each lead polar cell and translate that as above into a maximal rotation angle. We find an average maximal rotation of 39.1° with a range of (32°,43°).

Switching often occurs near gaps between nurse cells, which implies that the heterogeneous environment impacts behaviors. This is visualized in Fig 4B at the end of the simulation with both polar cell centers falling behind the average cluster movement. In other simulations, we saw more or less rotation depending on the forces applied to the system. We conclude that these four forces can produce shuffling of cell positions within the cluster. This does not rule out the possibility that molecular dynamics, such as cell communication or receptor saturation, play a role. However, our data suggest that the forces in our model are sufficient to cause the cluster to rotate as it translocates forward.

### Migratory speed and border cell number

Six to eight border cells and two anterior polar cells arise in the majority of wild type egg chambers. When too few border cells are specified, migration fails and affected egg chambers cannot be fertilized and give rise to offspring [3, 43]. Often when too many border cells form, they fail to detach and migration is also disrupted [3]. While JAK/STAT signaling levels influence how many motile cells are specified [3, 51], it is currently not known if the optimal number is determined by the minimal biophysical forces required to complete the movements. However, it is known that the cluster must not migrate too slowly, or else it will not arrive in time for the beginning of the next stage of oogenesis.

**Fig 5:**
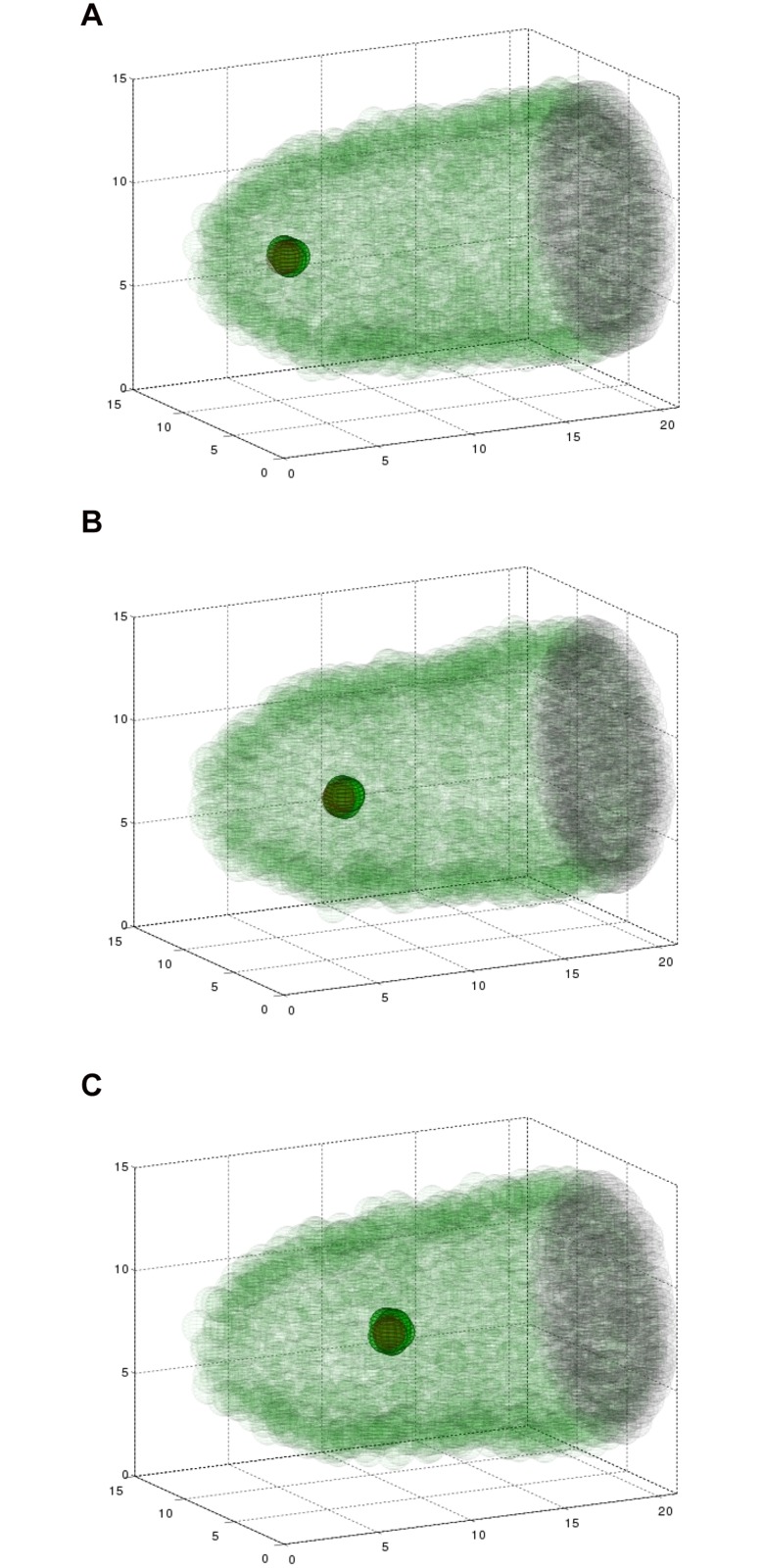
Simulations with four, six, and eight border cells at the same time point (*t* = 1.8 hours) during migration. The cluster with four border cells (A) has moved significantly less distance than the cluster with six (B) or eight (C) border cells.

We were interested to see how altering the number of border cells affected the behavior of the system. Specifically, we wondered if decreasing the number of migratory cells would slow the cluster down, because there would be a smaller total migratory force generated by the cluster and the two non-motile polar cells may be exposed, creating drag on the movement. Alternatively, it was possible that the smaller size of the cluster might actually result in it migrating faster, because it can fit between the nurse cells more easily. This question depends on the balance of forces; the repulsive forces from the nurse cells are pushing back on the migratory cluster as it pushes forward. We computed the model with identical parameters except for varying the number of border cells. We examined eight border cells, six border cells, or four border cells (with two polar cells in all cases) to observe how the forces between the cluster and its surroundings evolve, and how this alters overall behavior. Fig 5 shows the result of the simulation at the same time point for each of these three cases. At a mid-migration time point, the cluster with four border cells had not migrated as far as the cluster with six border cells, and the cluster of eight border cells migrated farthest. This suggests that additional motile cells help in translocating the polar cells to the destination of the oocyte.

We computed the real world time that the cluster took to migrate from the start of the simulation, with the cluster in the follicular layer, to the time that the cluster reached the oocyte. This is shown in Table 2. We observe that the clusters with six and eight border cells, migrated in 5.6 and 3.9 hours, respectively. This is within the feasible amount of time that the clusters have to migrate in the biological system [19, 20]. Over several simulations (N = 10), the six border cell cluster only varied with a standard deviation of about 0.5 minutes, so we conclude the level of stochasticity used here does not greatly affect the migration time. We observe that the cluster of cells with four border cells migrates slower than the clusters with six and eight border cells. The cluster with just four border cells took nearly five times as long to complete the migration than the cluster with eight border cells. *In vivo* egg chamber developmental progression, which results in a much larger oocyte, obscures analysis of migration past about 6 hours.

**Table 2:** The total time and relative time taken for computational clusters of 4, 6 and 8 border cells to complete migration.

Num. border cells	Migration time	Rel. migration time
4	18.9 hours	4.846
6	5.6 hours	1.423
8	3.9 hours	1

## Discussion

The process of collective cell migration that occurs during Drosophila oogenesis is a highly regulated, complex system. Using our mathematical model and numerical implementation, we can study how the forces between interacting cells balance to allow a cluster of motile and non-motile cells to migrate as a collective. We have demonstrated that the fundamental forces of adhesion, repulsion, migration and stochasticity in our model are sufficient to observe many features of the biological system. We posit that the forces of adhesion and repulsion are necessary to accurately model this behavior of the system: without the balance of these forces, the computational or biological system would not maintain its structural integrity, and would either collapse into itself (if the repulsion force was not existent), or would break apart (if the adhesive force was not holding it together). We have used our conceptual framework to show that the four key forces are sufficient to cause rotation of the migratory cluster, or cell-position-switching, in this system. Our analysis suggests that stochasticity is necessary to achieve pronounced rotation of the cluster. While more complex molecular relay mechanisms may contribute to the tumbling behavior, it appears to be an emergent property of the intercellular forces. We postulate that the cluster turns in part due to gaps between nurse cells, which prevent local cell-cell adhesions in that region.

*In vivo* genetic experiments show that STAT signaling establishes the number of motile cells and it maintains perdurance of movement. Mutations that reduce STAT signaling result in fewer motile cells and failure of collective migration. However, in these cases it is difficult to distinguish whether the delay in migration is a result of fewer cells, or a direct consequence of reduced signaling. Our simulations suggest that when too few border cells have been recruited, the time it takes for the cluster to traverse the egg chamber greatly increases as a result of insufficient biophysical forces. Mutant egg chambers with excessive STAT activity specify additional motile cells. Although larger clusters move faster in the simulations, in biological experiments, very large clusters tend to disaggregate into smaller groups or single cells, making these results hard to compare. A future research interest is to integrate more molecular signaling data into the biophysical model in an effort to recapitulate additional *in vivo* behaviors.

A unique aspect to this model is the use of aggregated IMCs to define heterogeneous tissue types. This model is capable of capturing the dynamics of a diverse cell population, and models the larger nurse cells by aggregating multiple IMCs together using increased adhesive forces. This aspect was important to mimic the *in vivo* movements of border cell clusters. A benefit of our IMC model construction is the flexibility of the initial condition specifier. This flexibility allows a researcher to place any cell type in any three-dimensional domain. This could be used to investigate other aspects of oogenesis, such as how asymmetry in the initial locations of border cells in the epithelium affects cluster formation. Additionally, the ability to specify a heterogeneous cell population at any location in space means that the implementation could be modified for other systems in which understanding the forces between cells is of interest, such as modeling the movement of epithelial cells in the intestine, the motility of metastatic cells, or other systems in which cells migrate as a group.

## Supporting Information

S1 Movie Time-lapse movie of border cell migration. Example of a live-imaging movie of wild-type border cell migration within a cultured egg chamber, corresponding to the still images in Fig 1B. The motile cells are marked in green by expression of Slbo-life-Act-GFP. The oocyte, which autofluoresces, is towards the bottom right. The nuclei of all cells, including the large, polyploid nurse cells, are seen in blue from the Hoescht DNA label. At time 0, the border cells have already clustered (top left) and begun moving towards the oocyte. In this example, the border cells reached the oocyte border by 3.5h.
(AVI)

S2 Movie Close-up movie shows motile cells rotating about the polar cells. Example of a time-lapse movie of wild-type border cell migration within an egg chamber, corresponding to the still images in Fig 1C. The edges of the border cells are marked in green by expression of Slbo-driven membrane tethered-GFP. The oocyte is out of view but towards the right. For details see Fig 1C.
(AVI)

S3 Movie Simulating the model results in collective migration. A time course from a simulation showing six border cells (solid green), two polar cells (within the border cells), the epithelium (transparent green), and the oocyte (black, right) over the course of three hours. Fifteen nurse cells are situated inside the egg chamber but are not plotted so the dynamic border cells can be observed. Motile cells can be seen to change relative positions, and movement towards the oocyte is non- uniform in velocity. For more details, see Fig 3.
(MOV)

S1 Appendix Determining the migratory direction in 3D. A brief calculation of the migratory direction in three dimensions.
(PDF)
