# Supplementary material for: A Mathematical Model of Collective Cell Migration in a Three-Dimensional, Heterogeneous Environment
Source: PLoS One. 2015 Apr 13;10(4):e0122799. doi: 10.1371/journal.pone.0122799 (PMC4395426; doi:10.1371/journal.pone.0122799)
Supplement: S1 Appendix — A brief calculation of the migratory direction in three dimensions. (PDF) [file pone.0122799.s004.pdf]

Supporting Information to: A mathematical model of collective cell migration in a three-dimensional, heterogeneous environment

David P. Stonko[1], Lathiena Manning[2], Michelle Starz-Gaiano[2], Bradford E. Peercy[1,*]

1 Department of Mathematics and Statistics, University of Maryland Baltimore County, MD, USA

2 Department of Biological Sciences, University of Maryland Baltimore County, MD, USA

∗ Corresponding Author: bpeercy@umbc.edu

S1_Appendix: Determining the migratory direction in 3D

To calculate the direction of IMC migration in three dimensions, we project the chemical gradient onto the plane that is orthogonal to the axis of interaction. This gives the direction of migration for the migratory cell. The interacting non-migratory cell experiences the same magnitude force, but in the opposite direction. We compute this force by using the Gram-Schmidt process on the plane that is orthogonal to their interaction to obtain an orthonormal basis for this plane. Then, the gradient of the chemical gradient is projected onto this plane.

In the two-dimensional system it was trivial to compute the direction of migration because in this case we have two points in this two-dimensional space. Thus, the vector that connects the center of two IMCs, d, only has two orthogonal vectors. Therefore, $d_{i,j}^{\perp}$ is a one dimensional subspace of $\mathbb{R}^2$ and $d_{i,j}^{\perp} \cdot d_{i,j} = 0$, so $\text{Proj}_{d_{i,j}^{\perp}} \nabla f = \alpha d_{i,j}^{\perp}$, where $\alpha = (d_{i,j}^{\perp} \cdot \nabla f)/(d_{i,j}^{\perp} \cdot d_{i,j}^{\perp})$. The three-dimensional formulation of the migratory force is also given by (4) in the main text, but is calculated differently because in three-dimensions, $d_{i,j}^{\perp}$ is a two-dimensional subspace of $\mathbb{R}^3$. Here $z_1 \cdot d_{i,j} = 0$, and $z_2 \cdot d_{i,j} = 0$ where $\{z_1, z_2\}$ is an orthogonal basis for $d_{i,j}^{\perp}$, so $\text{Proj}_{d_{i,j}^{\perp}} \nabla f = \alpha_1 z_1 + \alpha_2 z_2$ where $\alpha_1 = (z_1 \cdot \nabla f)/(z_1 \cdot z_1)$, and $\alpha_2 = (z_2 \cdot \nabla f)/(z_2 \cdot z_2)$.

In our model, we use the Gram-Schmidt process to construct z_1 and z_2. First, we compute the distance in the x, y and z directions between IMCs i and j. Call these distances, d_x, d_y, and d_z, respectively. Without loss of generality, let $d_x > d_y > d_z$. Then, define the vectors v_1 and v_2 to be

$$v_1 = [-d_y \ d_x \ 0],$$
$$v_2 = [-d_z \ 0 \ d_x],$$

chosen this way to ensure numerical stability. Now, take v_1 to be one of our basis vectors, z_1. Then find the orthogonal vector, which will be the second basis vector, z_2 by calculating α,

$$\alpha = \frac{v_1 \cdot v_2}{v_2 \cdot v_2}.$$

The non-zero size of this denominator is insured by the way that we chose v_1 and v_2 previously. Now, the second basis vector is given by

$$z_2 = v_2 - \alpha v_1.$$

Thus, we have orthogonal vectors z_1 and z_2 that define the plane that is orthogonal to d. We use these to compute the migratory force, which is given by Equation (4) in the main text.
